# Genetic and molecular regulation of chilling requirements in pear: breeding for climate change resilience

**DOI:** 10.3389/fpls.2024.1347527

**Published:** 2024-04-26

**Authors:** Gilad Gabay, Moshe A. Flaishman

**Affiliations:** ^1^ French Associates Institute for Agriculture and Biotechnology of Drylands, The Jacob Blaustein Institutes for Desert Research, Ben-Gurion University of the Negev, Sede-Boker, Israel; ^2^ Institute of Plant Sciences, Volcani Research Center, Rishon Lezion, Israel

**Keywords:** pear, QTL, dormancy, budbreak, chilling requirement

## Abstract

Pear (*Pyrus* spp.) is a deciduous fruit tree that requires exposure to sufficient chilling hours during the winter to establish dormancy, followed by favorable heat conditions during the spring for normal vegetative and floral budbreak. In contrast to most temperate woody species, apples and pears of the Rosaceae family are insensitive to photoperiod, and low temperature is the major factor that induces growth cessation and dormancy. Most European pear (*Pyrus Communis* L.) cultivars need to be grown in regions with high chilling unit (CU) accumulation to ensure early vegetative budbreak. Adequate vegetative budbreak time will ensure suitable metabolite accumulation, such as sugars, to support fruit set and vegetative development, providing the necessary metabolites for optimal fruit set and development. Many regions that were suitable for pear production suffer from a reduction in CU accumulation. According to climate prediction models, many temperate regions currently suitable for pear cultivation will experience a similar accumulation of CUs as observed in Mediterranean regions. Consequently, the Mediterranean region can serve as a suitable location for conducting pear breeding trials aimed at developing cultivars that will thrive in temperate regions in the decades to come. Due to recent climatic changes, bud dormancy attracts more attention, and several studies have been carried out aiming to discover the genetic and physiological factors associated with dormancy in deciduous fruit trees, including pears, along with their related biosynthetic pathways. In this review, current knowledge of the genetic mechanisms associated with bud dormancy in European pear and other *Pyrus* species is summarized, along with metabolites and physiological factors affecting dormancy establishment and release and chilling requirement determination. The genetic and physiological insights gained into the factors regulating pear dormancy phase transition and determining chilling requirements can accelerate the development of new pear cultivars better suited to both current and predicted future climatic conditions.

## 1 Introduction

Pear (*Pyrus* spp.) belongs to the *Rosaceae* family and the subfamily Pomoideae. The *Pyrus* genus is exceptionally diverse, encompassing 22 primary species with thousands of cultivars. *Pyrus* spp. is widely accepted to divide into three major groups: the European pear (*Pyrus communis* L.), Asian sand pear (*Pyrus pyrifolia* (Burm.) Nak.), and Chinese white pear (*Pyrus bretschneideri* Rehd) (Bell et al., 1990). Cultivated pears are diploids (2n = 34) with 17 chromosomes (Wu et al., 2013; Chagné et al., 2014; Gabay et al., 2018). Pears are economically significant deciduous fruit trees thriving in temperate climates (Nishio et al., 2016). They are cultivated across six continents, with substantial production in temperate regions like China, the United States, Italy, Argentina, and Spain, where ideal growing conditions, especially sufficient chilling during the winter, exist (Wu et al., 2013). The diverse climatic regions in which pear species can thrive highlight their remarkable adaptability and potential for growth in both warm and cold climates.

In temperate regions, perennial plants have developed a dormancy mechanism to survive under severe cold temperatures and frost (Lang et al., 1987; Bielenberg et al., 2008). The tree also accumulates sugars and starches, which act as energy reserves during the dormant period. The dormant period suspends tree growth, allowing them to survive harsh winter conditions. Their metabolic activity slows down significantly, and they are less susceptible to damage from freezing temperatures (Faust et al., 1997). In general, dormancy is characterized by the bud meristem being unable to resume growth under favorable conditions, such as heat exposure (Rohde and Bhalerao, 2007). Dormancy can be divided into three separate states: paradormancy, where external factors regulate and maintain dormancy, often related to apical dominance; endodormancy, regulated by chilling accumulation within floral and vegetative buds, which is the primary focus of this review; and ecodormancy, where environmental factors, mainly temperature-related, signal the bud to exit dormancy after sufficient chilling accumulation (Erez and Lavee, 1971; Lang et al., 1987; Erez et al., 1988, 1997).

Fruit tree breeding is a protracted process fraught with challenges such as lengthy juvenile periods and self-incompatibility. The long juvenile period in pears (<7 years) complicates the identification of genes governing vital agricultural traits (Visser, 1964; Hackett, 1985; Freiman et al., 2012). Consequently, the evolution of fruit quality traits in pears typically occurs when trees are at least 7 years old and, at times, even later. Thus, traditional pear breeding often spans 13 to 15 years. However, some traits, such as disease resistance and chilling requirements (CR) for vegetative growth, can be assessed early in a tree’s life (Montanari et al., 2016; van de Weg et al., 2018; Bell, 2019; Zurn et al., 2020).

To evaluate chilling units’ (CUs) accumulation in a specific growth region, several models have been proposed. The dynamic model is often used, especially in Mediterranean regions and other warm places such as California, which are characterized by warm winters. This model considers the negative effect of elevated temperatures during winter and the duration and intensity of exposure to cold temperatures (Fishman et al., 1987; Erez et al., 1988). In the context of climate change, CU accumulation is decreasing constantly, significantly impacting fruit productivity and quality (Campoy et al., 2019). Consequently, there is a growing demand for cultivars with low chilling requirements (CR). For that reason, many studies aiming to define the chilling requirements of cultivars from different Rosaceae species were conducted previously (Ruiz et al., 2007; Wenden et al., 2016; Ruiz et al., 2018; Gabay et al., 2018; Parkes et al., 2020) and in other taxonomic groups such as olive (Orlandi et al., 2004) and grape (Anzanello et al., 2018). The CRs of a specific cultivar or accession are evaluated by the number of CUs needed to induce at least 50% of vegetative budbreak after exposure to favorable conditions such as heat and day length (Hauagge and Cummins, 1991). Compared with other fruit crops, traditional pear breeding is both expensive and time-consuming due to extended seedling maturation periods and the production of numerous spines on juvenile trees, complicating harvest and orchard management (Brewer and Volz, 2019). Therefore, developing genetic markers for key agronomic traits is imperative to expedite the assessment of new cultivars, facilitating early-stage genotype evaluation (Wu et al., 2014; Zhang et al., 2021). Given that CR determination is a pivotal trait in numerous pear breeding initiatives (De Franceschi and Dondini, 2019), it is imperative to devise genetic tools facilitating the early-stage selection of seedlings possessing the requisite CRs for a particular region. Therefore, this review provides an update of genetic knowledge, encompassing genetic and physiological factors associated with dormancy in *Pyrus* species, to support forthcoming breeding endeavors and functional genomics studies in pear. These factors can be targeted for genomic editing or for identifying natural variations in specific genes, enabling the introgression of the required alleles. This process will facilitate the breeding of cultivars with lower CRs.

## 2 Chilling requirements and the effect of climate change

### 2.1 Pear growing in the era of climate change

The recent rise in temperature worldwide has led to a reduction in the CU accumulation (Campoy et al., 2011). Therefore, CR aspects have been studied in perennial deciduous trees in the context of climate change that resulted in decrease of the exposure to CUs (Campoy et al., 2011; Gabay et al., 2017). To ensure high fruit quality and a sufficient yield, active growth following dormancy requires accumulation of a certain number of CUs during the dormant period (Gabay and Flaishman, 2019).

The European pear (*Pyrus communis*), like many other Rosaceae fruit tree species, enters dormancy in response to decreasing temperatures (Cooke et al., 2012). Endodormancy release hinges on the accumulation of CUs during winter. When pear trees are not exposed to sufficient chilling during the winter, the vegetative budbreak (VB) time is delayed (Gabay et al., 2017). Delay in VB time can result in insufficient vegetative growth, leading to the tree being unable to supply the required sugars and metabolites necessary for optimal fruit set and development, resulting in reduced fruit quality and yield losses (Elkins et al., 2007). To bridge the gap between the actual CUs accumulated during the winter and the CRs of a specific cultivar, the common agricultural practice is the use of growth regulators through chemical budbreak sprays (Erez, 1994; Keilin et al., 2007). In this way, farmers can induce the desired vegetative budbreak (VB) timing when chilling units are not fulfilled. The application of chemical budbreak agents facilitates consistent spring growth to support fruit development and has the potential to expand pear cultivation areas (Labuschagné et al., 2002b). However, environmental concerns are driving demand for fruit trees with lower CRs to find alternatives and reduce the use of chemicals with associated health risks that are currently being applied (Ubi et al., 2010; van Dyk et al., 2010; Celton et al., 2011). To decipher the mechanisms that control CRs, understanding the network of genetic and physiological processes that induces dormancy stage transitions is crucial. It allows for the implementation of management practices, such as artificial chilling techniques in regions with mild winters, to optimize tree health and fruit production.

European pear cultivars considered to have high-quality fruit, such as ‘Bartlett’ and ‘Bosc’, typically exhibit high chilling requirements (CRs), whereas those with low CRs, such as ‘Spadona’ and ‘Coscia’, often lack certain fruit quality traits (Flaishman et al., 2001; Stern et al., 2013). Consequently, there is a demand for high-quality pear cultivars that require low chilling unit (CU) accumulation. Additionally, due to climatic changes, it is assumed that some temperate regions currently suitable for pear cultivation may not be adequate in the coming decades.

### 2.2 Environmental and physiological factors influencing pear dormancy initiation and release

In numerous deciduous tree species, endodormancy entrance is induced by a reduction in day length (Horvath et al., 2003). However, in Malinae, a subtribe in the Rosaceae family that includes pear and apple, trees enter endodormancy when the temperature decreases in autumn, regardless of the day length (Anderson et al., 1986; Heide and Prestrud, 2005). CRs vary among cultivars and species, determining the CUs needed for VB in spring. When exposed to favorable conditions, represented by heat requirement (HR), dormant budbreak can occur, and vegetative growth resumes (Erez and Lavee, 1971; Lang et al., 1987; Cooke et al., 2012). Several studies in Rosaceae indicate that when trees are exposed to higher chilling units than their requirements, the heat requirements for flowering decrease (Couvillon and Erez, 1985; Citadin et al., 2001). When European pear trees were exposed to similar chilling units (CUs) and heat requirements (HRs), genotypes with lower CRs showed earlier VB time (Gabay et al., 2017). Hence, sufficient accumulation of chilling units shortens the favorable heat period needed for VB and accelerates VB time. Therefore, the VB date of pear trees that were exposed to similar conditions is a reliable indicator of a genotype’s CRs.

In regions with warmer climates, there is usually a lack of CUs and enough heat hours required to induce budbreak, whereas in colder climates, only the HR is a limiting factor (Allard et al., 2016). Therefore, when selecting a favorable cultivar for each climate region, both HRs and CRs should be considered. Another factor for cultivar selection would be the time of harvest when growers would like to market their product early in the season as they can receive better market prices for their fruits (Dirlewanger et al., 2012). Early fruit production can be associated with both CRs and HRs. Hence, a cultivar that requires low exposure to heat before bloom will produce fruit early in the season, or when the CRs are fulfilled faster, there are fewer HRs needed for floral budbreak. However, in almond (Rosaceae family), flowering time is mostly determined by the cultivar-specific CRs (Egea et al., 2003), and in apple, which is closely genetically related to pear, the heritability of HRs is low compared with CRs (Celton et al., 2011; Allard et al., 2016); thus, heat requirements are mostly environmentally driven. In a genetic study on sweet cherry *(Prunus avium*) aimed at identifying CRs and HRs associated with flowering date, no QTLs for HR were found, providing further support for the small genetic effect on HR (Castède et al., 2014). The stronger genetic effect of CRs on flowering time, which is highly correlated with VB time, compared with HR, was also found in apricot (Ruiz et al., 2007), sweet cherry (Alburquerque et al., 2008), and peach (Okie and Blackburn, 2008), emphasizing that in Rosaceae, CRs have more significant impact on dormancy break than heat requirements, and therefore, breeding efforts should be focused on CR determination.

Another factor that should be considered when selecting a cultivar for a specific region is that low chilling cultivars may be susceptible to late frost damage. Hence, early bloom in the spring followed by extreme cold exposure can result in damage to vegetative growth and fruit development (Dirlewanger et al., 2012).

Vegetative buds and floral buds differ in their physiological characteristics and their dormancy regulation. Due to “apical dominance,” the flower buds usually develop at the terminal position of spurs and can emerge from the lateral buds only on older shoots (Ito et al., 1999). The determination of bud destiny occurs in late summer or autumn. During this time, the organs in the buds develop based on their differentiation into floral or vegetative buds.

Flower buds in pear exhibit mixed buds consisting of both reproductive and vegetative buds (Banno et al., 1986; Ito et al., 1999; Saito et al., 2015). Several studies have identified similar factors between floral (Gao et al., 2021) and vegetative (Gabay et al., 2019) buds associated with dormancy regulation. Thus, there is a high correlation between floral and vegetative bud dormancy. Additionally, there is a strong link between vegetative budbreak and flowering time (Allard et al., 2016). Therefore, in this review, we will discuss both floral and vegetative dormancy in parallel.

Exposure to chilling and flowering date are correlated, indicating that temperature during the chilling period influences the flowering time. For example, early flowering was observed after winters with lower temperatures, whereas late flowering occurred in seasons with higher winter temperatures (Fadón et al., 2023). The CRs and HRs needed to induce dormancy break differ between floral and vegetative buds. However, there is a high correlation in CRs and HRs between vegetative and floral budbreak in the subfamily Pomoideae (Allard et al., 2016). Hence, for low CR cultivars, both floral and VB will occur earlier compared with those with high CRs. The budbreak of vegetative and flower buds occurs after fulfilling CRs followed by sufficient heat exposure. The CRs for floral buds are usually lower than for vegetative buds. In peach [*Prunus persica* (L.) Batsch], the time for floral budbreak was earlier compared with VB (Jiménez et al., 2010), and in Japanese pear, leaf buds require a higher CR for dormancy break relative to their floral buds (Takemura et al., 2013). In apple, the CR to complete endodormancy of vegetative buds is the limiting condition, rather than that of flower buds, which require less chilling exposure during the winter (Naor et al., 2003). These results indicate that the CRs in the Rosaceae family, specifically in pear, are higher for VB compared with floral budbreak. Therefore, determining the vegetative CRs is the major factor that should be investigated to ensure optimal vegetative growth, supporting sufficient fruit development and quality. The dormancy of vegetative buds is deeper and requires greater exposure to chilling for dormancy release. Early VB timing is significant since vegetative growth is needed to support tree growth and fruit development (Elkins et al., 2007), and therefore, a relevant CR determination for breaking endodormancy in pears should be estimated using the CUs needed for VB (Banno et al., 1986; Ito et al., 1999; Saito et al., 2015).

### 2.3 Mediterranean low chilling climatic zone for forecasting future climate changes

European pear (*P. communis*) cultivars are primarily bred in regions with adequate winter temperatures to satisfy CRs, unlike the Mediterranean climate (Zohary, 1997). Therefore, pear tree performance of traits related to fruit quality and yield, including flower development and fruit set, differs across environments and climatic conditions (Labuschagné et al., 2002a). For that reason, pear is commercially grown mainly in temperate zones such as Eastern Asia, North America, Western Europe, and South Africa; however, due to the rise in temperatures and according to climate model predictions, the current climate change will result in a reduction in CU accumulation in these regions (Campoy et al., 2011). Therefore, it is likely that in the coming decades many pear production areas will not meet the CRs of the commercial cultivars and will result in decreased yield and poor fruit quality.

The Mediterranean region exhibits a diverse array of microclimates, spanning from arid/semiarid to temperate and humid conditions. Several models were developed to estimate CU accumulation in deciduous trees in low-CU accumulation regions like the Mediterranean, where endodormancy release is influenced more by recent climate changes. In warmer climates, a widely employed model for assessing CU accumulation is the dynamic model designed for estimating CRs. This model considers the adverse impact of high winter temperatures on CU accumulation (Erez et al., 1988). Climate change may influence plants through direct or indirect changes in dormancy. Over the past few decades, multiple global and regional climate models have identified the Mediterranean as a “Hot-Spot” for forecasting future climate changes in temperate regions (Giorgi et al., 2001; Saadi et al., 2015; Gabay et al., 2021). Hence, the current growing conditions in the Mediterranean will be similar to the growing conditions in temperate regions several decades from now. Within this area, numerous regions are currently witnessing a simultaneous rise in temperatures and a decline in precipitation. This ongoing trend has the potential to significantly impact the behavior and distribution of plant species, subsequently altering the physiology and phenology of various agriculturally significant species (Margiotta et al., 2017; Ferlito et al., 2021).

**Figure 1:**
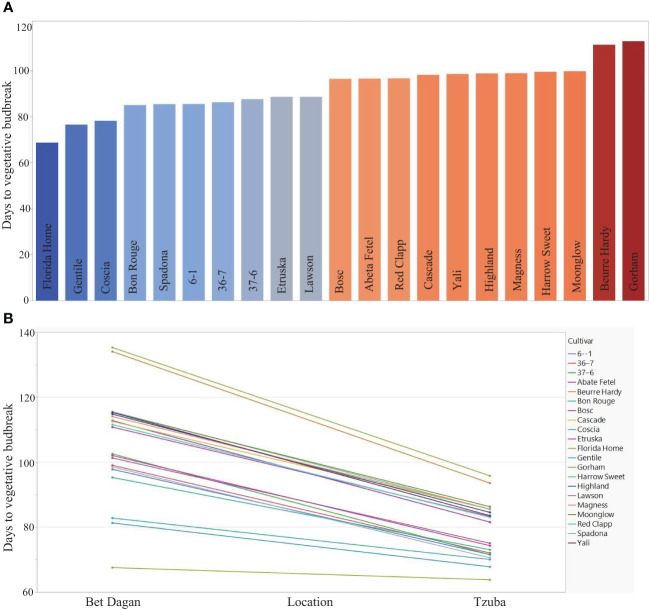
Examination of 21 Pyrus accessions in two locations differing in their chilling accumulation. **(A)** LS means of the average days to budbreak of the accession in two locations, ‘Bet Dagan’ = low chilling units’ accumulation and ‘Tzuba’ = high chilling units’ accumulation. **(B)** Interaction plot of Location × Cultivar of 21 accessions in two locations (Bet Dagan and Tzuba) indicates smaller standard deviation in Tzuba (8.69) compared with Bet Dagan (16.05). The average days to VB in Bet Dagan are 105.67, and in Tzuba, it is 78.9. The figure is modified from Gabay et al. (2018a).

In regions with low CUs, the variance of VB time is larger compared with regions that have sufficient chilling accumulation. The variance of natural vegetative budbreak in two different climatic regions in Israel showed remarkable differences between region with cold climate (“Tzuba”) and warm climate (“Bet Dagan”) (**Figure 1**). An efficient amount of carbohydrates is required for optimal fruit development (Elkins et al., 2007). The vegetative growth after dormancy release should be sufficient to provide these carbohydrates for fruit development. Therefore, a delayed VB will result in lower carbohydrate accumulation and can affect fruit quality and quantity. Since VB timing is a reliable indicator of CRs, it is important to grow the adequate cultivar in a specific region to avoid delaying VB time. In regions with low chilling unit (CU) accumulation, such as in Israel, the most widely grown cultivars are low-CR varieties, ‘Coscia’ and ‘Spadona’ (Stern et al., 2002). However, even for those cultivars, the CRs are not always fulfilled, resulting in delayed VB time that can affect productivity and fruit quality. Considering this, a current project is focused on performing a QTL analysis associated with abiotic (Gabay et al., 2019) and biotic (Gabay et al., 2021) stresses using an F_1_ population of European pear ‘Spadona’ (Low CRs cultivar) and ‘Harrow Sweet’ (High CRs cultivar) (SPD × HS). Currently, a fruit quality QTL study is being analyzed based on fruit quality trait measurements under a Mediterranean climate in Bet Dagan, Israel, for 2 years (2017–2018). We thoroughly assessed the fruit quality and reproductive and vegetative traits of this population. The outcomes of this study offer valuable tools for genomic selection, enabling more efficient pear breeding programs. The developed genetic markers that were identified in these studies are currently in use in pear breeding programs in Israel. This knowledge, particularly relevant in warm climate areas, can help accelerate breeding efforts, ultimately contributing to the development of low CRs improved pear varieties.

### 2.4 Pear genetic resources adapted to different chilling units’ exposure

The selection of appropriate varieties for a specific environment, considering factors like latitude, altitude, and slope exposure, plays a crucial role in determining the economic success of pear cultivation and other temperate woody species (Fadón et al., 2023). Pear is widely grown around the world, and it is cultivated in many regions with range of climatic conditions (Wu et al., 2013) and therefore there is a great variance in CRs for pear cultivars. Pear cultivars with varying CRs display distinct endodormancy characteristics during the accumulation of chilling units. For example, the high-CR cultivar ‘Suli’ (requiring 800 h–1,000 h of chilling) (Liu et al., 2012) exhibits a well-defined deep endodormancy phase. This stage is succeeded by a noticeable release of endodormancy following the accumulation of CUs. In contrast, the low-CR cultivar ‘Cuiguan’ (with a CR of <400 h), does not enter a deep endodormancy stage throughout the chilling accumulation process. These differences were demonstrated by the examination of floral budbreak rates after the exposure to different CUs. Thus, low-CR cultivars will show a higher rate of budbreak after the exposure to minimal CUs. High-CR cultivars are favored over low-CR cultivars for studying the biological changes occurring during endodormancy release, as they offer a clear distinction in endodormancy stages (Gao et al., 2021).

A valuable genetic source for low-CR stems from Japanese pear [*P*. *pyrifolia* (Burm. f.) Nakai]. Most of the commercial pear cultivars suitable for subtropical growth conditions (with low chilling unit accumulation) belong to *P. pyrifolia*. However, some of the fruit characteristics do not meet the European pear fruit quality. In contrast, most European pear (*P. communis*) cultivars require exposure of more than 800 chilling units and therefore are not well adapted to subtropical conditions. Furthermore, they may not be suitable for growth in temperate zones in the future due to the global rise in temperatures (Brewer and Volz, 2019). Therefore, there is an effort to introgress low CRs into European pear (*P. communis*) cultivars, which have high fruit quality with high CRs. Two examples include ‘Hood’ and ‘Florida home’, which require 250 chilling units (CUs) and are both derived from a cross between *P. communis* and *P. pyrifolia*. ‘Florida home’ was bred in the pear breeding program at the University of Florida (Sherman and Lyrene, 2002). Additional genetic sources were identified in a recent study, which aimed to evaluate the performance of important agriculture traits in response to different chilling units’ exposure by using the agroclimatic requirements of 61 European pear cultivars (Ferlito et al., 2021). This study emphasized the Sicilian pear germplasm, which is characterized by a low CR compared with another pear germplasm. These results suggest that Sicilian accessions can be used as a valuable genetic resource to introgress their low CRs to other commercial cultivars to restore traits such as yield and fruit quality. The chilling requirement was fulfilled after 700 h–900 h of cold exposure for most of the cultivars that were evaluated. Notably, the CRs for cv. ‘Gentile’ were fulfilled after being exposed to between 500 and 700 of CUs (Ferlito et al., 2021). In the study of Gabay et al. (2018a), 21 pear accessions were evaluated under two different locations that differ in the amount of CU accumulation (**Figure 1**). The CRs were evaluated using the LS means of the replicates in two different locations: ‘Tzuba’, in the Jerusalem Mountain, Israel, with high year average CUs (700 h-900 h) and ‘Bet Dagan’ in the coastal area of Israel, with low chilling year average hours (200 h–400 h). A notable genetic resource is cv. ‘Florida Home’, which showed the earliest VB date in both locations compared with the other germplasm (**Figure 1A**). The phylogenetic analysis revealed two groups corresponding to CRs, except for pear cv. ‘Florida Home’, which showed a distinct genetic background for CR determination due to its hybrid nature. ‘Florida Home’ is derived from a cross between European pear and Asian pear (Villalta et al., 2005). Interestingly, the variance of VB date in ‘Tzuba’ (high CU accumulation regions) was smaller than in Bet Dagan (low CUs) (**Figure 1B**), indicating the importance of low-CR cultivars in warm regions. Hence, the small differences in VB date that were observed in a location with high number of chilling units can result in smaller effect compared with location without a sufficient chilling unit accumulation (Gabay et al., 2018).

## 3 Dormancy molecular regulatory networks in pear

### 3.1 Pear genomic resources

The completion of pear whole-genome sequencing (Wu et al., 2013; Chagné et al., 2014) provided a valuable genomic resource. The availability of the pear genome has enabled the carrying out of genome-wide association studies (GWAS), greatly contributing to the development of genetic markers, and helping to reveal the genetic mechanisms underlying important agricultural traits (Kumar et al., 2017; Gabay et al., 2018, 2021; Zurn et al., 2020; Zhang et al., 2021). The genetic maps constructed for pears consist of 17 linkage groups (LGs), mirroring the 17 chromosomes in pears (Kumar et al., 2017; Gabay et al., 2018; Zhang et al., 2021).

Recently, a high-quality genome of the cultivated Chinese sand pear, ‘Cuiguan’ pear, a popular cultivar in south China with a low CR, was published (Gao et al., 2021). In this study, a multi-omics approach was conducted to explore the crucial genes responsible for releasing endodormancy induced by chilling exposure.

Apple (*Malus domestica*) and pear share substantial genomic similarities, making apple genetic studies a valuable resource for identifying genes and QTLs associated with vital agricultural traits. Apple genomic resources, including the availability of the apple sequenced genome, predated pear’s genomic resources (Chagné, 2015). Consequently, genetic markers identified in apple were subsequently applied in several pear studies (Celton et al., 2009). For instance, markers for genomic selection related to resistance to fire blight (Le Roux et al., 2012) and fruit softening (Costa et al., 2008) were initially discovered in apple and then found in homologue genomic regions in pear, indicating a high synteny level between the two species. However, some traits in pears are regulated by different genomic regions than in apples. For example, the most stable QTL for VB date was identified in apple on chromosome 9 (van Dyk et al., 2010; Celton et al., 2011; Allard et al., 2016), whereas the most stable QTL in pear was identified in LG8 (Gabay et al., 2018). Consequently, dissimilarities in marker locations or different genetic mechanisms for essential agricultural traits underscore the necessity for independent pear research and the development of pear-specific genetic resources (Yamamoto and Terakami, 2016), including pear-specific genetic markers (Gabay et al., 2018).

The advent of next-generation sequencing (NGS) has opened new avenues to explore the functions of genes associated with identified QTLs, such as whole transcriptome analysis (Bai et al., 2013; Gabay et al., 2019). Allelic variants of these genes serve as the foundation for developing genetic markers for marker-assisted selection (MAS) (De Franceschi and Dondini, 2019). Nevertheless, validating gene function in pear genetic studies is an uncommon and often impractical practice due to the protracted juvenile period, which can extend beyond 7 years before phenotype evaluation. Recent advancements in pear transformation technologies have created opportunities for functional gene studies in pear that were unavailable until recently, exemplified by a recent study validating the function of the *PbrSTONE* gene in regulating stone cell formation, a critical fruit quality trait in pear (Zhang et al., 2021). In addition, the development of transgenic juvenile free pear plants obtained by *PcTFL1* silencing provides an interesting tool to accelerate pear breeding (Freiman et al., 2012).

### 3.2 Pear genes associated with bud dormancy regulation

*Dormancy-associated MADS-box* (*DAM*) genes have emerged as key players in dormancy regulation, particularly in the context of plant bud development (Bielenberg et al., 2008). In the first study in pear of *DAM* genes, three *DAM* genes, referred to as *PpyMADS13* (1–3), were characterized through homologous cloning based on the peach *DAM* genes (Saito et al., 2013). Subsequent research revealed that pear *DAM* genes are regulated by C-repeat binding factors and abscisic acid (ABA)-binding factors (ABFs) and triggered by temperature decrease (Li et al., 2018; Yang et al., 2020).

Genome-wide analysis of the ‘Cuiguan’ pear genome unveiled the presence of five intact *DAM* genes, each designated according to its respective chromosomal location (Gao et al., 2021). The sequence similarity level was higher among proteins originating from the same species. The phylogenetic analysis revealed that the five pear DAMs formed a distinct cluster along with four apple SVP proteins and were grouped together with six peach DAM proteins. Among these, three *DAM* genes were found to be arranged in tandem located on chromosome 8, labeled as *DAM1*, *DAM2*, and *DAM3*, whereas two *DAM* genes on chromosome 15, which encode identical proteins, were named *DAM4-1* and *DAM4-2*. In both chromosomes, QTLs associated with CRs were identified before (Gabay et al., 2018). A robust collinear relationship between the peach ‘*EVG*’ locus and loci located on pear chromosomes 8 and 15 was identified by collinearity genomic analysis.

**Figure 2:**
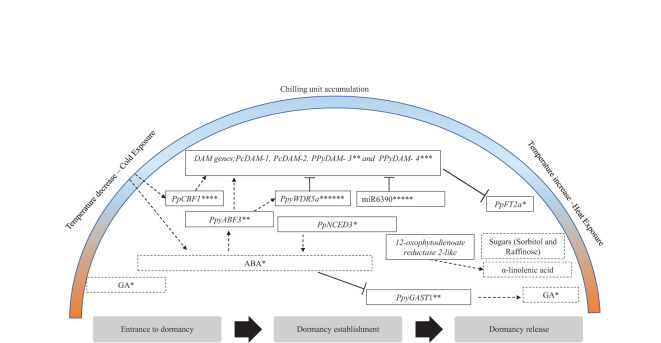
Key regulatory dormancy factors in pear and their suggested dormancy active period. Black frames indicate genes. Black dotted frame indicates hormones or metabolites. Arrows/bars indicate genes, hormones, metabolites, or environmental conditions suggested to induce/inhibit targets. The figure is modified from Gabay et al. (2018b), with additional information indicated with asterisks as follows: *Ito et al., 2021, **Yang et al., 2019, ***Gao et al., 2021, ****Li et al., 2019, *****Niu et al., 2016, ******Yang et al., 2023.

Using multiple biochemical approaches such as yeast one-hybrid (Y1H) assay and dual-luciferase assay, Li et al. (2019) showed the interaction between *PpCBFs* (transcription factors in the cold response pathway) and *PpDAMs* (**Figure 2**). This study suggests that *PpCBF1* plays a major role in responding to chilling and can regulate endodormancy by activating the *DAM* genes (**Table 1**) (Li et al., 2019).

**Table 1:** Recently published list of selected putative and validated pear genes associated with dormancy regulation.

Gene	Validation and identification type	Description	Expression peak time	Function	Subspecies	Reference
*PpNCED3*	Metabolic and expression analysis	ABA biosynthesis	Dormancy establishment	ABA regulation	*P. pyrifolia*	Ito et al., 2021
*PpyABF3*	Subcellular localization and protein interaction	ABA biosynthesis	Dormancy establishment	*PpyDAM3* gene activation	*P. pyrifolia*	Yang et al., 2020
miR6390	RNA-seq	DAM degradation	Dormancy release	*DAM-FT* interaction	*P. pyrifolia*	Niu et al., 2016
*CL9148*	RNA-seq	Dehydrin genes	Dormancy establishment	Cold response	*P. pyrifolia*	Liu et al., 2012
*PpyDAM-1*	Virus-induced gene silencing (VIGS) system	Dormancy-associated Mads box	Dormancy establishment	Endodormancy maintenance	*P. pyrifolia*	Gao et al., 2021
*PpyDAM-2*	Virus-induced gene silencing (VIGS) system	Dormancy-associated Mads box	Dormancy establishment	Endodormancy maintenance	*P. pyrifolia*	Gao et al., 2021
*PpyDAM-4*	Virus-induced gene silencing (VIGS) system	Dormancy-associated Mads box	Dormancy establishment	Endodormancy maintenance	*P. pyrifolia*	Gao et al., 2021
*PcDAM1*	QTL and RNA-seq	Dormancy-associated Mads box	Dormancy establishment	Endodormancy maintenance	*P. communis*	Gabay et al., 2019
*PcDAM2*	QTL and RNA-seq	Dormancy-associated Mads box	Dormancy establishment	Endodormancy maintenance	*P. communis*	Gabay et al., 2019
*PpyWDR5a*	Functional genomics	Epigenetic regulation	Dormancy establishment	DAM4 regulation	*P. pyrifolia*	Yang et al., 2023
*PpyGAST1*	Functional genomics	Hormone regulation	Dormancy release	ABA-GA interaction	*P. pyrifolia*	Yang et al., 2019
*PpFT2a*	Expression analysis	Ortholog of flowering locus T	Active growth	Flowering	*P. pyrifolia*	Ito et al., 2021
*PpCBF1*	Biochemical methods	Responding to chilling	Dormancy entrance	Activating DAM genes	*P. pyrifolia*	Li et al., 2019
*PpSOT2*	RNA-seq	Sugar synthesis		Sorbitol transporter	*P. pyrifolia*	Ito et al., 2012
*12-oxophytodienoate reductase 2-like*	QTL and RNA-seq	α-Linolenic acid pathway	Dormancy release	Biosynthesis of fatty acid	*P. communis*	Gabay et al., 2019

The candidate genes proposed in Gabay et al. (2018b) for the regulation of European pear vegetative bud dormancy align with previous research on *DAM* and *FT* genes in pear and apple (Bai et al., 2013; Mimida et al., 2015; Niu et al., 2016). Differentially expressed genes that underlie previously recognized major quantitative trait loci (QTLs) on LG8 were suggested as candidate putative genes for dormancy regulation in pear (Gabay et al., 2018), such as those related to the α-linolenic acid pathway, *12-oxophytodienoate reductase 2-like*, and suggesting that these genes play a critical role in dormancy phase transition (**Table 1**).

*PpyDAM3* expression regulates the content of ABA, a key hormone that regulates dormancy transition in deciduous trees, in pear (*Pyrus pyrifolia*) vegetative buds. The expression profile of pear *ABRE-BINDING FACTOR3* (*PpyABF3*) (**Table 1**) correlates with the expression of *PpyDAM3* during the dormancy phases, and the induced overexpression of *PpyABF3* upregulated the expression of *PpyDAM3* (Yang et al., 2020). The identified mechanism suggests that *PpyABF3* binds to the second ABRE (ABA-responsive element) in the *PpyDAM3* promoter to regulate its expression (Yang et al., 2020). In Gao et al. (2021), using an expression cluster analysis that corresponds to the different dormancy phases, several key putative genes associated with dormancy in pear were identified such as *ABRE-BINDING FACTOR* 2 (*ABF2*).

Another important gene related to ABA accumulation and correlated with ABA levels is *PpNCED3*. After the induction of endodormancy, the expression profile of *PpNCED3* was correlated with ABA levels, with expression lagging slightly behind ABA accumulation, suggesting that ABA accumulation is being regulated by the gene (Ito et al., 2021). In addition, this study suggests that *PpFT2a* plays a role in dormancy regulation (**Table 1**), exhibiting a negative correlation with dormancy. The expression of *PpFT2a*, associated with flowering time, is upregulated during flowering and downregulated during dormancy (Ito et al., 2021). Genes related to epigenetic mechanisms and phytohormone biosynthetic pathways are discussed in sections 3e and 4a.

### 3.3 Transcriptomes and gene expression profile of pear buds during different dormancy phases

To characterize the gene network regulating pear dormancy entrance, maintenance, and release, several transcriptome studies were conducted at different dormancy stages (Liu et al., 2012; Bai et al., 2013; Gabay et al., 2019; Gao et al., 2021).

In a high-chilling-requirement cultivar ‘Suli’ (*Pyrus pyrifolia*), the expression of *DAM3* showed a continuous decrease starting in the fall and this reduction persisted until the release of endodormancy. In contrast, *DAM1*, *DAM2*, and *DAM4* exhibited upregulated expression from dormancy establishment, with expression levels peaking just before the release of endodormancy (Gao et al., 2021). The expression pattern of *DAM3* in ‘Cuiguan’ pear, a low-CR cultivar, closely mirrored the pattern that was observed in ‘Suli’ pear. However, *DAM1*, *DAM2*, and *DAM4* in ‘Cuiguan’ pear showed a decrease in expression shortly after the release of endodormancy, which differed from the expression patterns in ‘Suli’ pear. Furthermore, the expression of the DAM genes in the high-CR cultivar ‘Suli’ was much higher during the dormancy maintenance, suggesting that dormancy depth is weak in the low-CR cultivar compare with the high-CR one, which had a higher expression of the *DAM* genes.

In a previous study (Gabay et al., 2019), we conducted an in-depth analysis of European pear (*Pyrus communis* L.) vegetative buds across various dormancy phases using RNA sequencing (RNA-Seq) and metabolic profiling. Our study involved two distinct *P. communis* cultivars with contrasting CRs: ‘Spadona’ (SPD), a low-CR cultivar, and Harrow Sweet (HS), a high-CR cultivar. *PcDAM1* and *PcDAM2*, putative orthologs of *PpDAM1* and *PpDAM2* (Asian pear), showed a higher expression at the entrance and the beginning of the dormancy stages, followed by a decrease in their expression at the end of dormancy and during VB. The strong correlation between the expression profiles of the *DAM* genes to dormancy transition stages suggests that endodormancy maintenance requires high *DAM* gene expression levels. Limited changes in *DAM* expression during endodormancy might be associated with shallow dormancy in low-CR cultivars and higher expression in high-CR cultivars (Gao et al., 2021).

Cluster analysis in Gao et al. (2021), using expression data from pear floral buds during dormancy phases, revealed several candidate genes showing similar expression patterns to *DAM* genes. These genes exhibited upregulation at dormancy entrance and increased expression during dormancy maintenance, followed by a decrease toward dormancy release, suggesting a correlation between their expression and dormancy stage transitions. Various transcription factors such as *NACs* (*NAC10*, *NAC40*, and *NAC88*), *MYB108*, *TCP12*, *ZAT12*, and *ABF2* were identified, along with enzymes like protein phosphatase 2C, sorbitol dehydrogenase, and E3 ubiquitin-protein ligase, within this cluster. Gene Ontology (GO) term enrichment analysis indicated their involvement in processes such as porphyrin-containing compound metabolic processes, tetrapyrrole metabolic processes, and pigment metabolic processes. Another gene cluster showed increased expression throughout the chilling accumulation period and included genes related to sugar biosynthesis such as fructokinase and sucrose synthase (Gao et al., 2021).

In another RNA expression study, a potential mechanism of dormancy regulation by miR6390 was proposed (Niu et al., 2016). According to this model, *PpDAM* blocks *PpFT2* expression to maintain dormancy. Upregulation of miR6390 results in the degradation of DAM products, removing inhibition of *PpFT2* and triggering floral budbreak.

In a transcriptome study of dormant buds of ‘Suli’ pear (*Pyrus pyrifolia*), a dehydrin gene (*CL9148*) was identified as a possible regulator of dormancy entrance by exposure to cold temperatures. Dehydrin genes are activated by ABA and CBF proteins and are activated by cold temperatures. *CL9148* showed differentially higher expression during the transition from endo to eco-dormancy followed by a decrease toward dormancy release (Liu et al., 2012).

Genes related to ABA and gibberellin biosynthesis were identified in the RNA-seq analysis of Japanese pear (*Pyrus pyrifolia* Nakai) (Bai et al., 2013). These genes were downregulated during dormancy break. Additionally, *aminocyclopropane-1-carboxylate synthase* (*ACS*), encoding an enzyme in the ethylene biosynthesis pathway, was upregulated toward dormancy release.

### 3.4 Validation of the functions of dormancy-related genes

The functions of the *DORMANCY-ASSOCIATED MADS-BOX* (*DAM*) genes inhibiting floral budbreak in pear buds was confirmed by employing a virus-induced gene silencing (VIGS) system (**Table 1**). A significantly higher budbreak percentage was observed in DAM-silenced dormant ‘Suli’ pear buds compared with the control buds was observed (Gao et al., 2021). Similarly in apple, silencing of *MdDAM1* and *MdDAM4* expression eliminates terminal bud formation and dormancy induction, which showed a similar phenotype to the ‘*EVG*’ mutant phenotype in peach (Moser et al., 2020). Another *DAM* member, *MdDAMb*, overexpression caused floral budbreak repression in apple (Wu et al., 2017). This indicates that the silencing of *DAM* genes likely facilitated the release of endodormancy, possibly through the induced expression of growth-related genes, such as *EXPA1*, which was previously correlated with endodormancy release in ‘Suli’ pear (*Pyrus pyrifolia* White Pear Group) (Yang et al., 2019).

### 3.5 Epigenetic control of pear dormancy

Gao et al. (2021) suggested that epigenetic modifications play a crucial role in regulating *DAM* genes during prolonged exposure. The ChIP-seq data showed that H3K4me3 and H3ac (histone modifications) were positively associated with *DAM* expression, whereas H3K27me3 (another histone modification) is negatively associated with *DAM* expression in kiwifruit and Prunus species (Leida et al., 2012; Vimont et al., 2020). In peach, chilling exposure triggers multiple epigenetic changes, including those involving 21-nucleotide small RNAs and non-coding RNAs (Zhu et al., 2020). These epigenetic alterations are inversely correlated with *DAM* downregulation. Additional expression analysis showed that *NAC88*, a gene related to cold-induced transcription factors, correlated with the increase in H3K4me3 levels during exposure to chilling (Gao et al., 2021). Y2H library screening revealed that *PpyABF3* (an ABA-responsive gene) interacts with *PpyWDR5a*. Functional studies of *PpyWDR5a* overexpression results in a higher level of histone modification in *DAM4*, leading to growth cessation of pear calli, whereas silencing *PpyWDR5a* (VIGS) increases budbreak percentage. These results suggest that *PpyWDR5a* regulates the expression of *DAM4* (Guak and Fuchigami, 2001; Zheng et al., 2015; Wang et al., 2016; Yang et al., 2023).

## 4 The metabolite and hormonal control of pear dormancy

### 4.1 Phytohormones

Phytohormones play a critical role in controlling the transition between different dormancy stages, triggered by both internal signals and environmental cues (Wang et al., 2016; Tuan et al., 2017; Li et al., 2018). Abscisic acid (ABA) is a well-studied phytohormone that plays a major role in the regulation of dormancy in deciduous trees. Several studies revealed the correlation between the abscission of the leaves during the autumn (i.e., entrance to dormancy) and changes of the ABA content level (Guak and Fuchigami, 2001; Zheng et al., 2015; Wang et al., 2016). These studies suggest that ABA represses bud–meristem activity. Therefore, elevated levels of ABA promote entrance to dormancy, and when the ABA level decreases, there is no longer cessation of growth induced by ABA, leading to dormancy release. The ABA signal induces the entrance into dormancy and helps to maintain the dormancy stage in pear (Li et al., 2018); hence, when a high level of ABA is induced, the tree will remain under dormancy even when they are exposed to favorable heat conditions. The profile of ABA content levels was measured during different dormancy phases and characterized as follows: an increase in levels in response to exposure to cold temperatures at dormancy entrance, followed by continuously increasing levels toward dormancy release, and then a decrease after dormancy break. A rise in ABA amount was identified again before blooming in pear (Ito et al., 2021). This suggests that the ABA content level may not be a prerequisite for the dormancy release; instead, high level of ABA can reflect deep dormancy phase in pear (**Table 2**). In Gao et al. (2021), phytohormone levels were measured in floral buds exposed to artificial chilling treatment and compared with buds under natural conditions. ABA content was notably high during the treatment that induced dormancy entrance and dormancy establishment, followed by a gradual decrease. This pattern mirrored the changes observed in ABA levels under natural conditions. Additionally, the changes in the ethylene precursor ACC displayed a contrasting trend to the ABA content, suggesting that ethylene might play a role in negative control of both the induction and release of endodormancy (**Table 2**). Furthermore, the levels of jasmonates, including jasmonic acid (JA) and jasmonoyl-isoleucine (JA-Ile), tended to gradually decrease throughout the chilling accumulation period (Gao et al., 2021). A recent study found that jasmonic acid is negatively correlated with vegetative growth, specifically branch growth of pear trees, and suggests that genes related to the jasmonic acid pathway play a critical role in branching regulation (Cheng et al., 2023). These studies indicate the importance of JA in response to chilling exposure and the regulation of vegetative growth cessation, as well as the resumption of growth after the dormancy break (**Table 2**).

**Table 2:** Selected metabolites and hormones associated with dormancy regulation.

Factor	Validation and identification type	Description	Peak time	Function	Subspecies	Reference
α-Linolenic acid	Transcriptome analysis and metabolic profiling	Fatty acid	Dormancy release	Membrane metabolite composition	*P. communis*	Gabay et al., 2019
Gibberellic acid	Transcriptome analysis and metabolic profiling	Hormone	Dormancy entrance	Growth control	*P. pyrifolia*	Ito et al., 2021
Abscisic acid	Metabolic analysis	Hormone	Dormancy establishment	Dormancy maintenance	*P. pyrifolia*	Ito et al., 2021
Ethylene	Transcriptome analysis and metabolic profiling	Hormone	Dormancy release	Growth control	*P. pyrifolia*	Gao et al., 2021
Jasmonic acid	Transcriptome analysis and metabolic profiling	Hormone	Dormancy entrance	Growth control	*P. pyrifolia*	Gao et al., 2021
Raffinose	Metabolic profiling	Sugar	Dormancy release	Cold response and growth resumption	*P. communis*	Gabay et al., 2019
Sorbitol	Metabolic profiling	Sugar	Dormancy release	Cold response and growth resumption	*P. pyrifolia*	Ito et al., 2012

Gibberellic acid (GA) biosynthetic-related genes were previously reported to be upregulated during dormancy and the entrance to dormancy, suggesting that GA has a role in the induction and maintenance of dormancy in Rosaceae (Bai et al., 2013). In a more recent study, GA content levels (GA_4_) were measured during dormancy entrance and dormancy in pear (*Pyrus pyrifolia* Nakai) and found to be high during the entrance to dormancy to the endodormancy establishment stage (Ito et al., 2021). Additionally, they used heat treatment to delay endodormancy entrance and did not find a difference in GA_4_ in response to those treatments. These results suggest that GA_4_ is related to the active growth of shoots during late autumn and not as a dormancy regulator. However, since dormancy is characterized by growth cessation, GA may play a negative role in dormancy regulation, and high levels of GA_4_ may reflect that the trees are not in deep dormancy (**Table 2**).

An interesting mechanism of the crosstalk between GA and ABA was suggested by Yang et al. (2019). Based on GA and ABA profiles coupled with transcriptomic data, they suggested that ABA represses the expression of *PpyGAST1* (**Table 1**) during dormancy. When the level of ABA content decreases, *PpyGAST1* can activate GA synthesis, which is necessary for dormancy release, perhaps by activating *PpyGA20OX1*, a key enzyme in the GA biosynthetic pathway. The sweet cherry homolog (*PavGA20ox-2*) was found to be associated with flowering time (Liu et al., 2023), which is correlated with chilling requirements (Egea et al., 2003). Furthermore, the overexpression of *PpyABF3* in pear induces the upregulation of *PcGA2OX1* (Yang et al., 2023). Loss of function induced by VIGS of *PpyABF3* and *PpyGA2OX1* enhanced budbreak and the upregulation of a cell-growth-related gene, *PpyEXPA1* (Yang et al., 2023). Multiple studies point out that the mechanism by which ABA regulates dormancy transition is related to intercellular communication.

Another important study related to dormancy identified that ABA could induce the synthesis of callose, resulting in the blockage of plasmodesmata, which are the intercellular channels through which cell communication is transmitted. When plasmodesmata is blocked, it disables the transport of growth and flowering factors such as FT1, which maintain the bud in the dormant phase (Tylewicz et al., 2018). Another possible GA-ABA interaction is that GA4 can restore the conductivity of the plasmodesmata channel by inducing β-1,3-glucanase to hydrolyze callose (Rinne et al., 2011). These related plasmodesmata mechanisms have not yet been studied and identified in pear.

### 4.2 Metabolites

There are limited studies on the genes that contribute to different metabolic pathways during dormancy in Rosaceae. Gabay et al. (2018b) introduces specific candidate genes linked to metabolic pathways, along with previously uncharacterized candidate genes of unknown function. Transcriptome analysis coupled with metabolic profiling of two European pear cultivars with distinct CRs revealed 22 differentially expressed genes (DEGs) associated with the α-linolenic acid pathway. JA is synthesized from α-linolenic acid (Eremina et al., 2016) and has been proposed to play a pivotal role in regulating dormancy break (Taniguchi et al., 2003). Importantly, a gene linked to *α-linolenic acid, 12-oxophytodienoate reductase 2-like* (LOC103967564) (**Table 1**), resides within the major QTL, associated with VB date, interval on LG 8 previously identified by Gabay et al. (2018a). A significant increase was observed in α-linolenic acid content toward the end of dormancy (**Table 2**). Six other unsaturated fatty acids, including linoleic acid, exhibited significant changes during dormancy. The fatty acid profile showed that α-linolenic acid, along with four other fatty acids, was characterized by low content throughout all dormancy stages and a sharp increase toward dormancy break. Notably, the level of fatty acids was directly correlated with chilling accumulation. This suggests a potential role for α-linolenic acid and the six other fatty acids, including lauric acid, linoleic acid, margaric acid, nonadecylic acid, palmitic acid, and stearic acid, in modulating membrane metabolite composition to facilitate VB.

Multiple studies have identified the correlation between sugar content levels and dormancy transition phases, suggesting a possible regulatory mechanism of sugars in dormancy development (Horikoshi et al., 2018). Sugar and carbohydrate content levels are suggested to play a role in cold tolerance (Sauter et al., 1996). In addition, sugar plays a major role in providing the energy supply needed for budbreak and growth at dormancy release (Ruan et al., 2010). A study of Japanese pear found that after a winter with low chilling accumulation, there is a decrease in carbohydrate composition (Gemma, 1995). A possible mechanism suggested by Ito et al. (2012) is the conversion of starch to sugar induced by exposure to cold temperatures. Two related enzymes, acid invertase (AI) and sucrose synthase (SuSy), were identified with the most significant activity, which paralleled dormancy entrance and release. Sugar metabolizing enzyme activity increased in buds after exposure to sufficient chilling units. Putative candidate genes associated with sugar, specifically sorbitol transportation in pear, were identified, such as the sorbitol symporter *PpSOT2*. These results suggest that sugar accumulation triggers and is necessary for floral budbreak and dormancy transition. The availability of sorbitol (**Table 2**), the most abundant sugar during dormancy break in pear is critical for floral budbreak (Ito et al., 2012). Significant changes were also observed in the Raffinose content levels (**Table 2**) in European pear at different dormancy stages (Gabay et al., 2019). Sugar content accumulation was higher toward dormancy establishment and VB, suggesting its role in protecting buds against drought as reported in apple (Falavigna et al., 2018), particularly following the transfer of trees from controlled conditions to natural conditions. This indicates that the accumulation of sugars serves as a signal for sufficient chilling accumulation, triggering budbreak, as previously reported in grape (Khalil-Ur-Rehman et al., 2017). Taken together, these studies suggest that sugar has two major roles during dormancy: first, to provide protection against cold stress, and later, when favorable heat conditions promote budbreak, sugars can provide the energy needed for the resumption of growth in both vegetative and reproductive systems.

A possible crosstalk between GA/ABA to sugar biosynthesis is not well-studied in pear. However, in Japanese apricot (*P. mume*), GA treatment induces the sugar metabolism pathway (Zhuang et al., 2015), and in grapevine, ABA inhibits sucrose transporters (Murcia et al., 2016). Additionally, ABA induces the upregulation of genes related to starch biosynthesis after entering dormancy, thereby promoting starch accumulation during dormancy in grapes (Rubio et al., 2019). Hence, a high level of ABA can maintain a high level of starch during dormancy. When ABA levels decrease after sufficient exposure to chilling units, the starch converts to sugars necessary for budbreak and growth resumption.

The metabolic profile of phospholipids showed accumulation in response to chilling exposure, with low-CR cultivar accumulating earlier compared with high-CR cultivar. Therefore, phospholipids may be accumulated in response to chilling as described in peach (Erez et al., 1997), and this accumulation occurs earlier in pear cultivars with lower CRs (Gabay et al., 2019).

## 5 Genetic factors associated with chilling requirements in pear

Most quantitative trait loci (QTLs) identified in pears are associated with fruit traits, disease resistance, and vegetative characteristics (De Franceschi and Dondini, 2019). It is essential to note that the localization of these loci varies based on testing duration, location, or populations studied, rendering genetic markers accurate for specific populations and locations. Moreover, these identified QTLs serve as potent tools for unraveling the genetic mechanisms underlying these traits (Wu et al., 2014; Zhang et al., 2021). The identification of genetic factors influencing CRs was first accomplished in peach (*Prunus*), a member of the Rosaceae family (Bielenberg et al., 2008). Within this context, *MADS-box* genes associated with dormancy regulation, including six *DORMANCY-ASSOCIATED MADS-BOX* (*DAM*) genes and a genomic region called the ‘*EVERGROWING*’ (‘*EVG*’) locus, were recognized for their roles in bud set, vegetative growth, and growth cessation (Jiménez et al., 2010). Subsequent quantitative trait locus (QTL) analysis using a substantial peach population identified a QTL associated with CRs within the same genomic region as the ‘*EVG*’ locus (Fan et al., 2010). This correlation between CRs and dormancy regulation is not unique to peach, as QTLs associated with both CRs, and bloom and VB date have been identified in other members of the Rosaceae family. In the *Prunus* genus (peach, apricot, and sweet cherry), the same QTLs were identified for CRs and bloom date, indicating a strong correlation between these traits (Dirlewanger et al., 2012). In previous genetic studies involving apple, which shares a high level of synteny with pear (Celton et al., 2009), QTLs associated with CRs were also identified (van Dyk et al., 2010; Celton et al., 2011; Allard et al., 2016). Notably, the sole QTL consistently found across different apple families was located on LG9, demonstrating its stability across various families, climate regions, and years (van Dyk et al., 2010; Allard et al., 2016).

In a recent study on pear, the QTL synteny between apple and pear was confirmed, using data derived from Genotyping By Sequence (GBS) (Gabay et al., 2018). In this genetic study of a segregating F_1_ population derived from a cross between low chilling cultivar (‘Spadona’) and a high-CR cultivar (‘Harrow Sweet’), two major QTLs were detected in the same genomic regions as in apple, LG9 and LG8, and they exhibited stability across different climates and years. Although pear and apple share high synteny (Celton et al., 2009), variations were observed in the genomic regions associated with CRs, with apple’s most significant QTL being detected on LG9, whereas pear’s major QTL was located on LG8 (Gabay et al., 2018). The detection of a new QTL associated with VB date on LG13 in pear was a noteworthy discovery, previously unreported in either pear or apple. These findings emphasize the significance of conducting independent genetic studies in pear and constructing high-resolution genetic maps to accurately pinpoint genomic regions associated with complex traits and determine variance values explained by these QTLs.

## 6 Marker-assisted selection using molecular markers and DNA variation

The reliability of a QTL for a specific trait and its utility in marker-assisted selection strategies depend on the QTL’s stability under various environmental conditions, locations, and genetic backgrounds (Ben Sadok et al., 2013). A study involving 21 European pear cultivars, categorized into high- and low-CR groups using “selective genotyping,” found significant molecular markers associated with VB date on the same genomic location of the identified major QTLs on LG8 and 9 (Gabay et al., 2018), suggesting that these regions control VB date in diverse pear genetic backgrounds.

G × E interaction is a significant factor that affects VB date in pear (Gabay et al., 2017). Pear genotypes respond differently to varying amounts of exposure to chilling units. There are also differences in the variance of VB between locations with varying chilling unit accumulations (**Figure 1**). Hence, the differences in low chilling unit exposure were larger compared with high chilling unit exposure. This trend highlights the importance of low chilling genotypes, especially in low chilling areas. G × E QTLs were identified on LG9 and LG5, with additional QTLs on LG8 and LG17 (Gabay et al., 2018). These QTLs indicate the presence of pear genotypes carrying useful genes or alleles, resulting in differences in VB dates across locations and climatic conditions. In a later study on sweet cherry (a member of the Rosaceae family), conducted in regions differing in their accumulation of chilling units (CUs), G × E QTLs were identified, providing further support for environmental interaction with genotype that affects flowering time (Branchereau et al., 2023). These QTLs could prove valuable in predicting genotypic stability across diverse environments, which is essential for matching CRs of Rosaceae and specifically pear cultivars to suitable growing regions. These QTLs can be used to develop molecular markers to define the suitability of a specific cultivar to specific climate conditions. Additionally, the selection of pear cultivars with adequate VB date traits is crucial for both warm and cold regions, particularly for low-CR pear cultivars susceptible to frost damage due to early budbreak caused by unexpected warm temperatures or by late frost in the spring (Dirlewanger et al., 2012).

Functional gene and QTL studies confirm the major role of the *DAM* genes in the regulation of dormancy phase transition (Gabay et al., 2018; Gao et al., 2021). The differential expression of the *DAM* genes between low CRs and high CRs was significant through different dormancy stages (Gao et al., 2021). Therefore, the identification of natural variation by DNA sequence or gene expression of the *DAM* genes can be utilized to develop genetic markers.

In conclusion, genetic studies in pear and the construction of high-resolution genetic maps have advanced our understanding of the genetic factors governing CRs in pear. The presence of QTLs associated with CRs and their stability across diverse conditions are valuable for future marker-assisted selection and enhancing the adaptation of pear cultivars to various climatic and environmental challenges (Gabay and Flaishman, 2019). The validated genes in this review (**Table 1**) can lead to the development of genetic markers for selecting low CR cultivars at an early developmental stage. Additionally, genes related to the biosynthetic pathways of metabolites and hormones, as presented in **Table 2**, should be prioritized for functional genomic studies.

## 7 Proposed model for dormancy in pear

Our proposed model of dormancy regulation considers shifts in the metabolic profile, phytohormones, and gene expression and functional validation of the selected studies presented in this review (**Figure 2**). These findings emphasize the influence of phytohormones, sugars, fatty acids, and *DAM* genes on dormancy regulation in pear and their response to cold exposure, chilling accumulation, and heat conditions. We postulate that, at the onset of dormancy, there is an accumulation of phospholipids and ABA alongside the accumulation of CUs, which might be necessary for sugar biosynthesis (Gabay et al., 2019) and to induce the upregulation of the *DAM* genes. ABA content levels peak at dormancy establishment and can indicate a deep dormancy phase in pear, whereas GA is an indicator of active growth (Ito et al., 2021). Dormancy release is characterized by a reduction in ABA levels and increased GA accumulation. These contradicting patterns, coupled with the expression of their biosynthetic pathway-related genes, suggest a possible crosstalk between these hormones (Yang et al., 2019). Subsequently, sugars accumulate and potentially act as a signal for sufficient chilling accumulation, paving the way for VB, as observed in grapes (Khalil-Ur-Rehman et al., 2017). The final phase of dormancy is characterized by an increase in fatty acids, leading to membrane changes reflected in a distinct metabolite composition, ultimately allowing budbreak to occur. Given the established role of plant hormones in pear dormancy (Bai et al., 2013; Li et al., 2018) and the fact that α-linolenic acid serves as a precursor to jasmonic acid (JA) (Eremina et al., 2016), We recommend investigating genes related to the suggested phytohormone biosynthesis pathway in future studies. Our focus in this model centered on genes underlying significant quantitative trait loci (QTLs) (Gabay et al., 2019) and genes with validated functions (Yang et al., 2019; Gao et al., 2021; Ito et al., 2021). The summary of the regulatory factors influencing dormancy release and establishment, as presented in this review, is illustrated in **Figure 2**.

## 8 Conclusions and perspectives

Over the past decade, extensive research has focused on the temperature-mediated regulation of bud dormancy, especially in model trees like poplar. However, our understanding of the regulatory mechanisms governing bud dormancy in pear vegetative and floral buds, along with their associated molecular mechanisms, remains limited. Flower buds require less chilling exposure compared with vegetative buds during the winter for dormancy release. However, the CRs for floral and vegetative buds are correlated. Therefore, determining the CRs for vegetative buds VB is critical for selecting a cultivar for a specific growing region (Naor et al., 2003). The genetic mechanisms that regulate the dormancy of both types of buds often involve similar genes that play a role in the dormancy transition mechanism in pear, such as the *DAM*- and *FT*-related genes (Gabay et al., 2019; Gao et al., 2021). However, to the best of our knowledge, no study has investigated the differences between the transcriptome profiles of vegetative and flower buds from the same tree of a specific cultivar. Such a study could be valuable for understanding the differences in dormancy transition phases between these correlated vegetative and reproductive organs. This may provide a broader toolbox to optimize pear growing under climatic disorders and drastic changes.

To unravel the intricate regulatory network involved in dormancy, powerful genomic and genetic approaches, such as quantitative trait locus analysis and genome-wide association studies (Gabay et al., 2018), are employed to identify the genetic regions responsible for natural variations in pear dormancy regulation. To validate the function of the candidate genes, there is a need to improve the functional genomics methods that are starting to be utilized in pear genetic studies (Gao et al., 2021; Yang et al., 2023). Additionally, studying trees cultivated under various climatic conditions proves valuable in comprehending both common and distinct mechanisms influencing dormancy in pears. The cultivation of deciduous fruit trees, including pear, in low-chilling areas allows us to observe the effects of global warming and warm winter conditions on dormancy release and the phenology of pear trees. Anticipated changes in fall, winter, and spring temperatures due to climate change are expected to influence the timing and intensity of growth resumption and flowering progression. This shift in climate could potentially limit suitable pear tree growing areas and impact well-established pear industries.

Confronted with these challenges, it becomes imperative to redouble efforts in establishing breeding programs in hot climate regions. The aim is to develop low-chill pear cultivars that not only exhibit high fruit quality but also possess sufficient storability. The knowledge gained from these efforts can be effectively harnessed in pear breeding programs targeting the development of new cultivars with lower CRs that align with current climate conditions.

## Author contributions

GG: Conceptualization, Data curation, Visualization, Writing – original draft, Writing – review & editing. MF: Writing – original draft, Writing – review & editing, Conceptualization, Data curation.
